# The accuracy of virtual setup in simulating treatment outcomes in orthodontic practice: a systematic review 

**DOI:** 10.1038/s41405-023-00167-3

**Published:** 2023-08-28

**Authors:** Benja Sereewisai, Rochaya Chintavalakorn, Peerapong Santiwong, Theerasak Nakornnoi, Siew Peng Neoh, Kawin Sipiyaruk

**Affiliations:** Department of Orthodontics, Faculty of Dentistry, Mahidol University, Bangkok, Thailand

**Keywords:** Orthodontics, Dental education

## Abstract

**Objectives:**

To evaluate the accuracy of virtual orthodontic setup in simulating treatment outcomes and to determine whether virtual setup should be used in orthodontic practice and education.

**Materials and Methods:**

A systematic search was performed in five electronic databases: PubMed, Scopus, Embase, ProQuest Dissertations & Theses Global, and Google Scholar from January 2000 to November 2022 to identify all potentially relevant evidence. The reference lists of identified articles were also screened for relevant literature. The last search was conducted on 30 November 2022.

**Results:**

This systematic review included twenty-one articles, where all of them were assessed as moderate risk of bias. The extracted data were categorized into three groups, which were: (1) Virtual setup and manual setup; (2) Virtual setup and actual outcomes in clear aligner treatment; (3) Virtual setup and actual outcomes in fixed appliance treatment. There appeared to be statistically significant differences between virtual setups and actual treatment outcomes, however the discrepancies were clinically acceptable.

**Conclusion:**

This systematic review supports the use of orthodontic virtual setups, and therefore they should be implemented in orthodontic practice and education with clinically acceptable accuracy. However, high-quality research should be required to confirm the accuracy of virtual setups in simulating treatment outcomes.

## Introduction

Orthodontic practice requires proper diagnosis and treatment planning to achieve expected treatment outcomes. However, especially in complicated orthodontic cases such as asymmetric extraction or multiple missing teeth, there tend to be more than one possible treatment options [1, 2]. These possibilities therefore should be simulated to predict their expected outcomes. A traditional approach of dental setup has been developed to simulate the orthodontic tooth movement by sectioning each tooth in a plaster model, moving them to favorable locations, and then positioning the moved teeth by wax [2–4]. However, this traditional dental setup requires considerable time and effort to complete the procedure.

Virtual orthodontic setups or tooth movement simulations have recently been adopted in orthodontics to overcome challenges of the traditional approach. Similar to other digital orthodontics, virtual dental setup can be considered as storage-space-friendly [5], damage-free [6, 7], and user-friendly [5, 7]. In addition, with the virtual setup, a number of possible treatment plans for an orthodontic problem (e.g., extraction, interproximal reduction, or expansion techniques to gain space for crowding elimination) can be simulated to visualize their results [8–10]. Virtual orthodontic setup can also undergo superimposition with the initial model, where the precise amount of tooth movement can be analyzed in each treatment option [11, 12]. Following the favorable characteristics of the virtual orthodontic setups, they should be considered as a replacement for traditional plaster model setups.

Not only the virtual orthodontic setup can be supportive for patient care, but it can also be in an educational aspect. As there is evidence that the visualization of tooth movement simulation could enhance a communication during the discussion of orthodontic treatment plans [9, 11, 13], it could support the discussion between orthodontic residents and their clinical advisors in discussing treatment outcomes of various orthodontic cases. Virtual setups can also be used for a case conference where orthodontists could present their treatment to their colleagues for educational purposes [7]. With the virtual setup, orthodontic patients with various craniofacial problems can be simulated, where orthodontic residents could gain experiences and improve their cognitive competence in safe environments through computer-generated tooth movement [14]. Consequently, virtual setup can play an important role as a technology-enhanced learning tool in orthodontic education.

Albeit the advantages of the virtual orthodontic setup, its precision and reliability seem to be a point of concern [15, 16]. There could be a number of errors at any steps, including obtaining the digital model, either by intraoral or plaster model scanning [17, 18], importing files to various simulation software [7, 19, 20], performing virtual tooth segmentation [21–23], moving teeth according to optimal treatment plan in different software [24, 25], and the measurement techniques of tooth movement [12, 26]. Although there had been several studies evaluating the reliability and accuracy of virtual orthodontic setup, this concern has not been yet comprehensively reviewed. As virtual orthodontic setup and simulation software have potential for clinical and educational purposes, this systematic review was conducted to evaluate their accuracy in simulating treatment outcomes.

## Materials and methods

### Review design

A systematic review was selected to evaluate the accuracy of digital orthodontic setups by comparing their tooth movement simulation to outcomes of actual treatments or manual setups, with a purpose of determining whether or not they should be used for clinical and educational purposes. This review methodology allows researchers to analyze a group of information on an interesting topic. Systematic reviews require the application of scientific strategies to minimize potential bias in reviewing all relevant evidence on a selected topic, to critically appraise and synthesize into a single comprehensive report [27–29]. The scientific procedures of systematic review are involving seven consecutive stages [27], including: (1) identify research questions or purposes; (2) identify research protocols; (3) systematically search relevant literature according to the inclusion and exclusion criteria; (4) extract data into organized categories; (5) assess quality of all the retrieved literatures; (6) collate, summarize, and report results; and (7) interpret results. Hence, systematic reviews can provide valid conclusions as well as valid evidence base for a selected topic.

### Search strategy

The systematic search was performed across five electronic databases, which were PubMed, Embase, Scopus, ProQuest Dissertations & Theses (PQDT) Global, and Google Scholar. Gray literature was also expected from Google Scholar to cover orthodontic virtual setups wherever possible. The reference lists of identified articles were screened for relevant literature. The iterative searches were performed to adjust the search strategy and search terms to assure the robustness of this review [30]. The search terms were developed according to PICO approach and was detailed in Table 1. However, search terms for comparison finally were not included in order to ensure that as many as relevant articles would be identified. The last search of this systematic review was performed on 30 November 2022.

**Table 1:** Search strategy used for the systematic search.

P - Population	orthodontics OR orthodontic OR orthognathic OR “tooth alignment” OR “tooth movement”
I - Intervention	simulation OR “virtual set-up” OR “virtual setup” OR “digital set-up” OR “digital setup” OR “virtual treatment plan”
C - Comparison	“manual set-up” OR “manual setup” OR “clear aligner” OR “fixed appliance”
O - Outcomes	reliability OR reliable OR accuracy OR accurate OR validate OR validity OR precision OR precise OR error* OR discrepanc* OR superimpos*

### Inclusion and exclusion criteria

All types of empirical study regarding the accuracy of virtual setup in orthodontics published from January 2000 to November 2022 were eligible for this review. However, they were excluded if they were not relevant to orthodontic tooth movement and if they were not reporting outcomes of accuracy assessment. They were also excluded if they were not available in full-text or in English. These inclusion and exclusion criteria were presented in Table 2.

**Table 2:** Inclusion and exclusion criteria for article selection.

Inclusion criteria	Exclusion criteria
• Any types of empirical studies • Articles studying the accuracy of virtual setup in orthodontics • Articles published between January 2000 and November 2022	• Articles not relevant to orthodontic tooth movement or treatment outcome simulation • Articles not available in full text • Articles not available in English • Articles not reporting outcomes of accuracy assessment • Articles of expert opinion, case report, case series, or any types of literature review including meta-analysis

### Article selection

Systematic searches and article screening were independently conducted by two researchers (BS and KS). The eligibility of the pre-identified articles was confirmed by the two researchers (BS and KS) independently after a screening of titles, abstracts, and full-text. Any disagreements on the article selection between the researchers were resolved by discussing and consulting with the third researcher (RC) by considering inclusion and exclusion criteria.

### Risk of bias assessment for included articles

The strength of systematic reviews depends on the recruitment of high-quality studies, so assessing the quality of included articles is essential. The two researchers (B.S. and K.S.) independently assessed the quality and the strength of evidence of included articles using Swedish Council on Technology Assessment in Health Care (SBU) and Center for Reviews and Dissemination (CRD) [31], which could be graded into three levels of evidence as shown in Table 3. Similar to the article selection process, in the event of disagreement between the two researchers, the quality assessment was discussed with the other researcher (R.C.). The evaluation of included articles would reflect the level of evidence of this systematic review according to the protocol of SBU and CRD (Table 4). This tool was user-friendly and suitable for a fundamental appraisal of grading evidence in a systematic review [32]. Therefore, it was used as a checklist for determining the quality of articles included in this review.

**Table 3:** Criteria for grading of assessed studies.

Grade A–High value of evidence	All criteria should be met: • Randomized clinical study or a prospective study with a well- defined control group • Defined diagnosis and endpoints • Diagnostic reliability tests and reproducibility tests described Blinded outcome assessment
Grade B—Moderate value of evidence	All criteria should be met: • Cohort study or retrospective case series with defined control or reference group • Defined diagnosis and endpoints • Diagnostic reliability tests and reproducibility tests described
Grade C—Low value of evidence	One or more of the conditions below: • Large attrition • Unclear diagnosis and endpoints • Poorly defined patient material

**Table 4:** Definitions of evidence level.

Level	Evidence	Definition
1	Strong	At least two studies assessed with level “A”
2	Moderate	One study with level “A” and at least two studies with level “B”
3	Limited	At least two studies with level “B”
4	Inconclusive	Fewer than two studies with level “B”

A risk of bias assessment of included articles was performed using the Cochrane Collaboration’s tool [33], which was ‘Risk of Bias In Non-randomized Studies of Interventions (ROBINS-I)’. This tool could be used widely as a domain-based assessment, rather than focusing on only clinical treatment interventions in the evaluation of healthcare experimental research like other tools. ROBINS-I allowed researchers to assess a risk of bias of each included articles in seven domains, which were biases due to “confounding, selection of participants into the study, classification of intervention, deviations from intended interventions, missing data, and measurement of outcomes, and selection of the reported result”. All included articles were evaluated whether their risk of bias was low, moderate, high, or unclear. The assessment outcomes would inform whether the evidence included in this systematic review was robust or not, by considering the quality of included articles in terms of research methodology and report.

### Data extraction and synthesis

The data from included articles were extracted and synthesized in the following categories: type of virtual setup, research objectives, methodology, outcome measurement, key findings, author conclusion, and risk of bias assessment (Tables S1–S3). The data synthesis was then performed using a narrative approach, where the themes of this systematic review would be constructed from the extracted information.

## Results

### Articles identified from the search

**Fig. 1:**
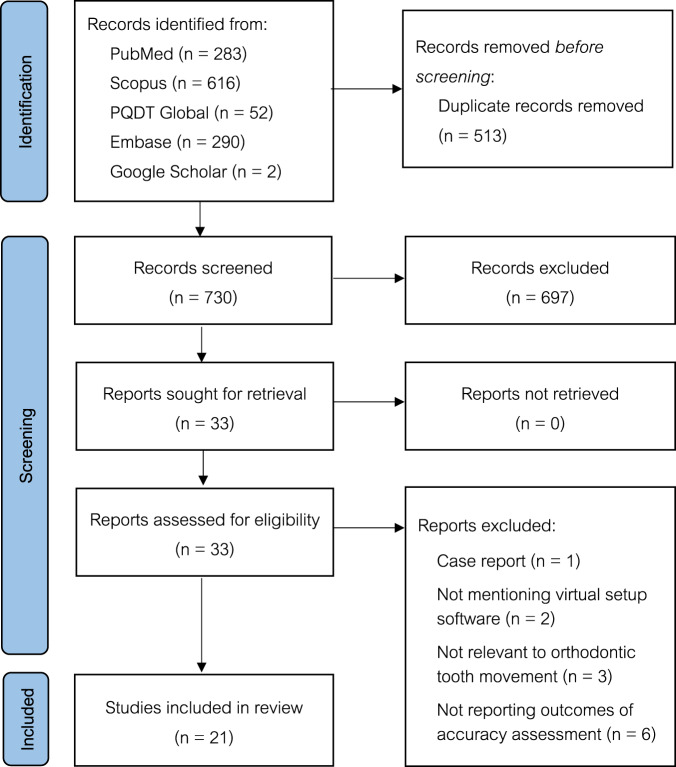
PRISMA 2020 flow diagram of the article selection process.

As presented in the PRISMA flow chart for study selection (Fig. 1), the electronic searches revealed 1241 articles from the four databases (PubMed = 283, Scopus = 616, Embase = 290, and PQDT Global = 52), and two studies were identified from Google Scholar. There was no additional research identified from the reference lists of included articles. After 513 duplicates were removed, 730 titles and abstracts were screened with consideration of the inclusion and exclusion criteria. Following the initial screen, 33 articles were selected for a full-text review, and 12 of them were excluded due to being a case report, no virtual setup software mentioned, no comparison of virtual setup and other techniques, being not relevant to orthodontic tooth movement, or no outcomes of accuracy assessment reported. Consequently, 21 full-texts were included in this systematic review.

### Quality of the articles included in this review

When evaluating the strength of included evidence with SBU, there were three article of prospective clinical trials that could be considered as a high value of evidence (Grade A) [34–36]. The other included articles appeared to be of moderate value of evidence, as they were retrospective studies. Therefore, the overall level of evidence of this systematic review was considered as strong, with three article of Level ‘A’ evidence and the other studies of Level ‘B’. According to ROBINS-I assessment, all included articles were evaluated as low or moderate risk of bias for all domains, so all of them were interpreted as moderate risk of bias. Although no included research was considered as high quality (low risk of bias), the quality of included evidence was not considered as problematic, as nearly all of them were non-randomized studies. Therefore, the risks of bias were mostly from confounding factors of research designs, such as different setup providers [35, 37, 38], no mention of a setup provider [35, 39–42], varying degree of malocclusion at beginning of the treatment [39, 42–45], and presence of any additional mechanics [44–47]. Also, the researchers who assessed the outcomes were not blinded in several articles [25, 34–38, 40–45, 47–52]. Test-retest reliability was not performed to confirm the reproducibility and reliability of the measurement in five articles [25, 34, 40, 41, 45]. Only one article performed an interrater reliability to confirm the consistency between the two assessors [44].

### Study design of included articles

Most of the included studies were non-randomized retrospective studies (*n* = 17), with exception of one prospective randomized clinical trial [34], two prospective non-randomized clinical research [35, 36], and one retrospective randomized research [39]. The sample size of included articles varied from ten to ninety-four samples, presenting various types of orthodontic problems were included ranging from mild to severe malocclusion. Out of twenty-one studies included in this systematic review, there were three articles comparing the treatment outcomes between manual and virtual setup [37, 39, 48]. The outcomes of virtual setup and actual treatment were compared in 18 articles, where the accuracy of virtual setup in clear aligners were evaluated in ten studies [25, 34–36, 38, 40, 41, 43, 49, 50] while eight research evaluated its accuracy in fixed orthodontic appliances [42, 44–47, 51–53].

### Virtual setup software used in included articles

There were a number of software used for virtual setup as reported in the included articles. ClinCheck appeared to be the most popular software used in six articles [25, 34, 35, 40, 43, 49], followed by OrthoAnalyzer in five studies [37–39, 44, 47], SureSmile in three papers [42, 45, 53], and Maestro 3D [50, 51]. Other tooth movement simulations were 3Txer [48], Airnivol [36], Flash [25], OrthoDS 4.6 [41], eXceed software [52], and uLab [46], where each of them was included in only an article. In addition, there were four studies, implementing cone-beam computed tomography systems (CBCT) to tooth simulation software, in order to provide more precise information with a reference to the face and skull of patients [44, 47, 49, 51].

### Outcome measurements

To measure the accuracy of tooth movement simulation, the treatment outcomes of virtual setup were compared with manual setup or actual treatment, where the differences between two approaches were compared in terms of linear intra-arch, interarch dimensions, and angular dimension. The comparisons were performed by digital software measurement [36–38, 40, 46, 50, 53], manually handed measurement [39, 48], or superimposition with a best-fit method [25, 34, 35, 41–45, 47, 49, 51, 52]. Seven included studies have clearly defined the threshold values of tooth movement discrepancies between virtual setup or actual treatment in reference to the American Board of Orthodontics (ABO) model grading system [25, 34, 42, 43, 45, 47, 52]. Thus, clinically significant discrepancies were set at over 0.5 mm for linear movements and over 2 degrees for angular movements in these articles. However, Smith et al. [53] set a discrepancy of 2.5 degrees of tooth tip and torque to be clinically acceptable variation for tip and torque.

### Accuracy of virtual setup

The accuracy of tooth movement simulations can be categorized into three groups, depending on the interventions that virtual setup was compared with, which were: (1) the accuracy of virtual setup in simulating treatment outcomes compared with manual setup, (2) the accuracy of virtual setup in simulating treatment outcomes of clear aligner treatment, and (3) the accuracy of virtual setup in simulating treatment outcomes of fixed appliance treatment.

#### The accuracy of virtual setup in simulating treatment outcomes compared with manual setup

There were three articles comparing treatment outcomes between virtual and manual setup [37, 39, 48]. Two articles supported the accuracy of tooth movement simulation using OrthoAnalyzer and 3Txer software [37, 48], as virtual and manual setups provided comparable measurements of treatment outcomes. However, there was an article reporting that there were statistically significant differences in tooth movement simulation between the virtual and conventional setups [39], where the printed virtual setup was less accurate than conventional setup with small accuracy differences from printing technology, tooth collision and software limitations. The data of the included articles in this group were extracted in Table S1.

#### The accuracy of virtual setup in simulating treatment outcomes of clear aligner treatment

There were ten articles comparing treatment outcomes between virtual and aligner treatment [25, 34–36, 38, 40, 41, 43, 49, 50], where the patients included in all of these studies were non-extraction and non-surgical cases. ClinCheck was the most popular software used for clear aligner prediction [25, 34, 35, 40, 43, 49], and other virtual setups were Flash [25], OrthoAnalyzer [38], OrthoDs 4.6 [41], Airnivol [36], and Masetro 3D [50]. There appeared to be discrepancies between tooth movement simulations from these virtual setups and actual treatment outcomes.

All included studies demonstrated statistically significant differences between predicted and achieved tooth positions [25, 34, 35, 40, 43, 49]. The accuracy seemed to be higher in linear dimensions compared to angular dimensions [25, 34] and in transverse direction compared to vertical and sagittal directions [35, 49]. The most precisely predictable tooth movement was tipping movement especially in maxillary and mandibular anterior teeth, followed by torque and rotation [36, 38, 41, 50]. Sorour et al. [25] also compared ClinCheck and Flash and found no clinically statistically differences in accuracy and efficacy between Invisalign or Flash aligner systems. The data of the included articles in this group were extracted in Table S2.

#### The accuracy of virtual setup in simulating treatment outcomes of fixed appliance treatment

There were eight articles comparing treatment outcomes between virtual setups and fixed appliance treatment [42, 44–47, 51–53]. The tooth simulation software used in these articles included SureSmile [42, 45, 53], OrthoAnalyzer [44, 47], uLab [46], Maestro 3D [51], and eXceed [52]. The patients in these studies had more severe orthodontic problems than those of the comparison between virtual setups and clear aligner treatment, as five articles considered extraction cases [42, 45, 46, 52, 53], while three articles evaluated orthodontic treatment combined with orthognathic surgery [44, 47, 51]. There was only one article reporting that an indirect bonding technique was performed for orthodontic bracket placement [45].

The degrees of accuracy were various depending on the software, tooth position, and types of tooth movement. SureSmile appeared to be more accurate in mesiodistal and vertical directions than buccolingual position, and there seemed to be clinically significant discrepancies in angular movements (tip and torque) of nearly all teeth [45, 53]. Its highest precision could be expected for translational and rotational movements of incisor teeth, where the accuracy decreased from anterior to posterior areas [42]. Research in OrthoAnalyzer also demonstrated the similar degree of accuracy to SureSmile. Although there were statistically significant discrepancies in tooth movement, clinically significance was not found, resulting its potential for treatment plan discussion [44]. However, it could be considered as less accurate in more complicated cases especially in rotational and translational directions [47]. Research in uLab [46], Maestro 3D [51], and eXceed [52] also presented statistically significant discrepancies in tooth movement simulation, however they could be used for the purposes of treatment planning and outcome visualization due to acceptable clinical discrepancies. The data of the included articles in this group were extracted in Table S3.

## Discussion

This systematic review was designed to include research published between January 2000 and November 2022. However, no identified article published between 2000 and 2012 was included in this review following the consideration of inclusion and exclusion criteria. During the period of 2013 to 2017, the research emphasized on comparing the accuracy of tooth movement simulations to manual setups [37, 48] and to actual treatment outcomes retrieved from fixed appliance treatment [42, 45, 51, 53]. The research focus had then moved to the comparison between virtual setups and clear aligner treatment during the period of 2018 to 2022, where seven out of ten articles were identified [25, 34–36, 40, 49, 50]. More recent publications had implemented CBCT superimposition to investigate root visualization and allow additional references from a skull [41, 44, 47, 49, 51, 53]. This implies the trend changes in the use of virtual setup over the 10-year period, which could be influenced by the current improvement and affordability of tooth movement simulation software.

The accuracy of virtual setups in simulating orthodontic tooth movement reported in the included articles can be considered as acceptable. The findings retrieved from those articles demonstrated statistical differences between virtual setups and actual treatment outcomes, but the discrepancies were clinically acceptable in non-extraction and non-surgery cases. The virtual setups tended to be more predictable in translation [25, 34, 44, 45, 47] and tipping movements [38, 41, 49, 51]. This could be a result from the flexibility of clear aligner materials, so they may have difficulties to control torque movement. The accuracy of treatment outcome simulation in clear aligners was greater in transverse prediction compared to vertical and sagittal directions [35, 49]. This could be due to orthodontic treatment planning where changes in arch width are generally minimized to aid in achieving stable treatment outcomes. Less accurate vertical and sagittal predictions could be a result of aligner thickness and improper anchorage control, respectively. In addition, due to the diversity in tooth movement methods of the included articles, the accuracy of virtual setups was categorized into three groups. However, the severity of malocclusions in treatment with fixed appliances tended to be more complicated than those treated with clear aligners. As tooth movement simulation for more complicated orthodontic problems could lead to more inaccuracies, there were difficulties in comparing their outcomes, especially between fixed appliances and clear aligners.

There appeared to be a number of factors making orthodontic tooth movement of virtual setups differed from actual treatment. As mentioned, only a few included articles employed virtual setups together with CBCT, so the movements of dental roots were not simulated in most of the studies. Therefore, unrealistic orthodontic tooth movements could be simulated, as surrounding tissues including biological limitations might not be considered [39, 48, 54]. In other words, less restrictions of tooth movements on computer simulation should be emphasized. Bone density and root morphology of the teeth could also affect orthodontic tooth movement [25, 36, 38, 43–45, 50]. The measurement extended to gum areas could also not be accurately assesses due to soft tissue distortion within virtual setups [37, 39]. In addition, following the digital segmentation, individually sectioned teeth of virtual setups appear to be smaller in mesiodistal width due to the hollowness of the inner proximal part of the model [48]. These limitations of virtual setups should be considered when performing tooth movement simulation.

Based on the findings in this systematic review, virtual setups should be implemented to simulate treatment planning in orthodontic practice. Tooth movement simulation can provide a chance for orthodontists to review their treatment plans with adequate precision in patients with mild to moderate malocclusions [34, 35, 38, 42–44, 46, 50–53]. However, an actual treatment outcome can differ from a simulated outcome due to a number of factors [25, 36, 38, 43–45, 50]. Patient compliance could also affect the treatment outcome [34, 36, 44, 53]. Therefore, orthodontists should acknowledge the limitations of the virtual setups when performing tooth movement simulation.

In addition to the advantages of virtual setups in clinical practice, they can be considered as supportive in orthodontic education. Virtual setup can provide safe learning environments for orthodontic residents to perform digital tooth movement and predict treatment outcomes, with or without supervision, in various orthodontic cases repetitively until they are competent for clinical practice. There is evidence of an increasing use of these tooth movement simulations in orthodontic education, where residents may use the virtual setups to present and discuss their treatment plans with clinical advisors [14, 55, 56]. Moreover, virtual setup could be used for a case conference where orthodontic professionals can discuss various cases with their colleagues [7, 11]. Therefore, with acceptable clinical discrepancies, virtual setups should be used for the purposes of education.

Most of the articles included in this systematic review are retrospective studies with no research was considered as high quality. In addition, there seems to be heterogeneity of research methodology of included articles, e.g., tooth simulation software, orthodontic appliance, severity of malocclusion, and outcome measurements, which could influence the accuracy of virtual setups. As virtual setup is an operator-dependent procedure, the research outcomes of included articles could be affected by different orthodontists or laboratory technicians who perform the tooth movement simulation procedure. Consequently, additional high-quality research with robust protocols should be required in order to enable meta-analysis to be performed to confirm the accuracy of virtual setup. Further research investigating the effectiveness and feasibility of virtual setups in either orthodontic practice or education should also be considered.

## Conclusions

The available evidence demonstrates the clinically acceptable accuracy of orthodontic virtual setups in simulating treatment outcomes, especially in cases with less complexity of tooth movement. Therefore, virtual setups are suitable to be implemented into both orthodontic practice and education, bearing in mind their limitations and discrepancies. However, due to the moderate risk of bias of all included article, high-quality studies with homogeneity of research and clinical protocols should be further required to confirm the accuracy and effectiveness of virtual setups in simulating treatment outcomes of different orthodontic problems.

### Supplementary information

Supplementary Information

## Data Availability

The data that support the findings of this study are available from the corresponding author, upon reasonable request.
