# Correction: The accuracy of virtual setup in simulating treatment outcomes in orthodontic practice: a systematic review

**DOI:** 10.1038/s41405-023-00169-1

**Published:** 2023-09-12

**Authors:** Benja Sereewisai, Rochaya Chintavalakorn, Peerapong Santiwong, Theerasak Nakornnoi, Siew Peng Neoh, Kawin Sipiyaruk

**Affiliations:** Department of Orthodontics, Faculty of Dentistry, Mahidol University, Bangkok, Thailand

**Keywords:** Orthodontics, Dental education

Correction to: *BDJ Open* 10.1038/s41405-023-00167-3, published online 28 August 2023

When originally published on 28 August 2023, the Data Availability Statement included the following sentence: ‘The data are not publicly available due to information that could compromise the privacy of research participants.’ However, as this is a Systematic Review (as opposed to primary research), no human participants were involved and therefore this sentence should have been omitted.

The correct Data Availability Statement reads in its entirety: ‘The data that support the findings of this study are available from the corresponding author, upon reasonable request.’

The authors apologise for any inconvenience caused.

