# Supplementary material for: The accuracy of virtual setup in simulating treatment outcomes in orthodontic practice: a systematic review
Source: BDJ Open. 2023 Aug 28;9:41. doi: 10.1038/s41405-023-00167-3 (PMC10462720; doi:10.1038/s41405-023-00167-3)
Supplement: Supplementary file 1 — Supplementary Information [file 41405_2023_167_MOESM1_ESM.pdf]

Table S1: Accuracy comparison between virtual setup and manual setup

Authors (Year)	Virtual setup software	Research objectives	Methodology	Outcome measurements	Key findings	Author conclusions	SBU	ROBIN-I
Barreto et al. (2016) [37]	OrthoAnalyzer	To examine the accuracy between virtual and manual setup at the end of orthodontic treatment	The measurements were performed on the scanned models using OrthoAnalyzer software (n=20)	- Intercanine width<br>- Intermolar width<br>- U/L arch length	None of the measures showed statistically significant differences.	Digital setups are as accurate as manual setups for diagnosing and treatment planning in orthodontic treatments.	B	Moderate
González Guzmán et al. (2019) [39]	OrthoAnalyzer	To compare 3D printed virtual setups with manual setups.	Printed virtual setup and manual setup models were measured using a digital caliper (n=10)	- Dimensional accuracy<br>- Overbite<br>- Overjet<br>- Dental arch length<br>- Intercanine width<br>- Intermolar width	- Most measurements showed higher values on manual setup compared to 3D printed setups, with overall differences were 0.24 and 0.14 mm for the upper and lower arches, respectively.<br>- Intercanine and Intermolar width did not show a statistically significant difference, but overjet and overbite did.	3D printed setups were not comparable to conventional setups. Only intra-arch measurements and dimensional accuracy showed good agreements.	B	Moderate
Im et al. (2014) [48]	3Txer	To compare the virtual and manual tooth setups in extraction cases.	The manual and virtual setups of samples with extracted upper and lower first premolars were simulated, and the printed models were analyzed using a digital caliper (n=10)	- Linear variables (arch width, arch length, arch perimeter, overjet, overbite)<br>- Angular variables (inclination, angulation, rotation)	The virtual setups showed smaller arch perimeters, overjet and overbite.	There seemed to be similar measurements between virtual and manual setups in intra-arch and interarch variables. However, adjustments for occlusal and interproximal contacts were required due to the collisions on the contacts.	B	Moderate

Table S2: Accuracy comparison between virtual setup and actual post-treatment outcome in clear aligner treatment

Authors (Year)	Type of virtual setup	Research objectives	Methodology	Outcome measurements	Key findings	Author conclusions	SBU	ROBIN-I
Al-Nadawi et al. (2021) [34]	ClinCheck	To compare the accuracy of virtual setups with actual outcomes of three aligner wear protocols:<br>1) 7-day changes<br>2) 10-day changes<br>3) 14-day changes	The posttreatment outcomes of the three groups of aligners were compared with virtual setups through digital superimposition (best-fit) using eModel version 9.0 software (n=80).	- Linear variables (mesiodistal, buccolingual, occlusogingival)<br>- Angular variables (inclination, angulation, rotation)	All linear discrepancies were not clinically significant (0.5 mm), while nearly all angular values were clinically significant (2°), where the 14-day group changes showed greater accuracy. There were discrepancies of upper and lower posterior intrusion (0.3 mm vs. 0.4 mm), upper posterior distal crown tip (2.88° vs 3.98°), upper posterior buccal crown torque (2.88° vs 4.48°), and lower posterior extrusion (0.2 mm vs 0.3 mm).	The group of 14-day changes were significantly more accurate, however the differences of accuracy did not exceed the threshold for clinical significance (0.5 mm/2°).	A	Moderate
D'Antò et al. (2022) [36]	Airnivol®	To evaluate the predictability of virtual setup with actual outcomes of clear aligner treatment.	Measurements of virtual setup with actual outcomes were performed by a single operator using VAM software (n=17).	- Inclination<br>- Angulation<br>- Rotation	Rotation of all teeth showed high accuracy, while tipping of second molars had the largest deviation (more than 4°).	There was a significant difference between virtual setup with actual outcomes of clear aligner treatment.	A	Moderate
Grunheid et al. (2017) [43]	ClinCheck	To evaluate the accuracy of Invisalign technology in predicting tooth positions considering tooth type	Actual treatment of non-extraction Invisalign treatment were superimposed with virtual setups using best-fit surface-	- Linear variables (mesiodistal, buccolingual, occlusogingival)	There were statistically significant differences between virtual setup and actual treatment for nearly all teeth except upper	There was high accuracy of ClinCheck to simulate actual outcomes in non-extraction Invisalign patients, although some measurements or	B	Moderate

Authors (Year)	Type of virtual setup	Research objectives	Methodology	Outcome measurements	Key findings	Author conclusions	SBU	ROBIN-I
		and tooth movement direction.	based registration (eModel version 9.0).	- Angular variables (inclination, angulation, rotation)	lateral incisors, canines, and first premolars.	treatment outcomes were different from the prediction.		
Lin et al. (2022) [49]	ClinCheck	To examine the accuracy of virtual tooth movement in ClinCheck with actual outcomes of invisalign treatment in Class II non-extraction patients by using digital models integrated into CBCT.	The initial and final digital models were integrated into CBCT and were then superimposed using voxel-based registrations to evaluate the changes of dentitions and mandibular position (n=7).	- Horizontal variables (intercanine width, inter-first-premolar, inter-second-premolar, inter-first-molar width)<br>- Vertical variables (central incisor, first molar)<br>- Anterior-posterior variables (central incisor, first molar)	- The accuracy in the horizontal direction was above 80%.<br>- Incisors were slightly more occlusal than predicted with accuracy of 44.3%.<br>- The actual outcome of distalization was significantly less than virtual treatment with accuracy of 37%-57% varied by teeth.	Overcorrection for the intrusion of upper and lower incisors and the design of skeletal anchorage to facilitate arch distalization and anterior intrusion should be considered.	B	Moderate
Lione et al. (2022) [35]	ClinCheck	To examine the accuracy of virtual setups in simulating distal and mesial rotation of upper first permanent molars treated with Invisalign in Class II malocclusion.	Pre- and post-treatment digital models were compared with the actual outcome from ClinCheck (n=40).	- Henry's angle (H°)<br>- Mesiobuccal expansion<br>- Distobuccal expansion<br>- Mesiobuccal sagittal<br>- Distobuccal sagittal	- H° difference between virtual and actual outcomes was 1.1°.<br>- Transversal expansion showed high accuracy.<br>- Sagittal plane showed a discrepancy about 1 mm.<br>- The accuracy was 82% for molar derotation.	There was accuracy for the expansion of upper arch associated with the distal rotation upper of first molar.	A	Moderate
Lombardo et al. (2017) [38]	Ortho Analyzer®	To examine the predictability of F22 aligners in guiding teeth into the positions	Virtual setup and actual treatment models were compared using VAM software (n=16).	- Inclination<br>- Angulation<br>- Rotation	- The accuracy of virtual outcome was 73.6%.<br>- Tipping showed the most accuracy (82.5%),	Tipping movements had high predictability, especially at the premolars and molars, but the	B	Moderate

Authors (Year)	Type of virtual setup	Research objectives	Methodology	Outcome measurements	Key findings	Author conclusions	SBU	ROBIN-I
		planned using digital orthodontic setup.			followed by torque (72.9%) and rotation (66.8%).	lower canine rotation was unpredictable.		
Riede et al. (2021) [40]	ClinCheck	To examine the accuracy between virtual setups simulated in ClinCheck and Invisalign treatment in achieving upper arch expansion or contraction.	Two investigators analyzed and compared maxillary models of pretreatment model, posttreatment clinical model, and ClinCheck setup model (n=30).	- Thirteen transverse parameters<br>- Occlusal contacts	- There were significant differences between the virtual setup and treatment outcomes, in which transverse parameters were observed with accuracy ranging from 28%-57% varied by tooth type and measuring site.<br>- Intense occlusal contacts in virtual setups were less than actual outcomes.	- The accuracy in achieving the predicted transverse goals was 45%.<br>- The effectiveness of achieving clinically ideal contacts was 59.1%	B	Moderate
Sorour et al. (2022) [25]	- ClinCheck<br>- Flash	To evaluate the accuracy of virtual setups in predicting tooth movement for Invisalign and Flash aligners.	Pre-, post-treatment, and virtual setup models were compared using eModel Compare 8.1 software. (Invisalign: n=38; Flash: n=24).	- Linear variables (mesiodistal, buccolingual, occlusogingival)<br>- Angular variables (inclination, angulation, rotation)	- There were statistically and clinically significant differences in angular movements between virtual setup and treatment outcomes for ClinCheck and Flash.<br>- There were statistically but not clinically significant differences in Flash, with better accuracy for central incisor tip (1.3°) and	Both Invisalign or Flash aligner systems had statistically and clinically significant differences between virtual setup and treatment outcome in angular movement.	B	Moderate

Authors (Year)	Type of virtual setup	Research objectives	Methodology	Outcome measurements	Key findings	Author conclusions	SBU	ROBIN-I
					buccolingual canine movement (0.1 mm).			
Tepedino et al. (2018) [50]	Maestro 3D	To evaluate the accuracy of virtual setups in simulating anterior tooth torque in Nuvola aligner	Anterior teeth torque of 39 non-extraction orthodontic patients with Nuvola aligners were measured on digital models scanned by 3shape at pre- and post-treatment models as well as virtual setups.	Inclination of anterior teeth	No statistically significant difference was found for all the anterior teeth between predicted and achieved torque movements, with the mean differences less than 1°.	Virtual setups could simulate treatment outcomes of Nuvola aligner in achieving anterior tooth torque.	B	Moderate
Zhang et al. (2015) [41]	OrthoDS 4.6	To assess the accuracy of integrated three-dimensional digital models in anterior tooth movement using clear aligners.	Digital models were integrated with 3D CBCT, and the differences between predicted and achieved outcomes were compared with superimposition (n=32).	Crown and root position of maxillary and mandibular anterior teeth	- The discrepancies in maxillary and mandibular crown positions were 0.376 mm and 0.398 mm.<br>- The discrepancies in maxillary and mandibular root positions were 2.062 mm and 1.941 mm.	Crowns of upper and lower anterior teeth can be moved to predicted positions using clear aligners, but not for roots, because these appliances appeared to move teeth by tipping.	B	Moderate

Table S3: Accuracy comparison between virtual setup and actual post-treatment outcome in fixed appliance treatment

Authors (Year)	Type of virtual setup	Research objectives	Methodology	Outcome measurements	Key findings	Author conclusions	SBU	ROBIN-I
Baan et al. (2019) [44]	OrthoAnalyzer	To investigate the accuracy of virtual orthodontic setups with a new CBCT- based approach.	The virtual and post treatment digital models were fused with CBCT scans, and then aligned and measured by 2 interrater-validated observers (n=10).	- Linear variables (mesiodistal, buccolingual, occlusogingival)<br>- Angular variables (inclination, angulation, rotation)	There were small differences between the virtual setup and actual treatment outcomes in the translations (less than 0.45 mm ± 0.48-1.14 mm) but large in rotations, especially molars (less than 3.04° ± 3.31-10.26°).	The differences between virtual setups and actual treatments were clinically insignificant for diagnostic purposes, so treatment outcomes can be simulated using virtual setups.	B	Moderate
Müller-Hartwich et al. (2016) [42]	SureSmile	To evaluate the accuracy of virtual setups with SureSmile system.	Virtual setups were made with digital models after their completion of leveling. The virtual setups and actual treatment outcomes were best-fit-superimposed by GeoAnalyzer (n=26).	- Linear variables (mesiodistal, buccolingual, occlusogingival)<br>- Angular variables (inclination, angulation, rotation)	- Translational deviations in buccolingual and mesiodistal planes were 0.19 mm, also 0.21 mm in vertical plane.<br>- Rotational deviations were 2.12° for inclination, 1.77° for angulation, and 3.04° for rotation.<br>- The precision decreased from anterior to posterior.	There was clinically successful predictability in virtual setups with SureSmile process, especially for rotations and translations of incisors.	B	Moderate
Kim et al. (2017) [51]	Maestro 3D	To evaluate the accuracy of virtual tooth movement with surgical simulation and actual treatment outcomes.	The virtual setups and post-actual treatment outcomes were superimposed with a surface-based registration method using Rapidform XOV2 software (n=11).	Linear variables (Each landmark on central incisors, canine and first molars to the sagittal, horizontal, and coronal planes)	Differences less than 2 mm were found in most on all three planes between virtual setups and actual outcomes.	Virtual tooth movement with surgical simulation was accurate when compared with actual treatment outcomes, which can be used for diagnosis and planning although some landmarks showed discrepancy.	B	Moderate

Larson et al. (2013) [45]	SureSmile	To evaluate the effectiveness of SureSmile to achieve the tooth position simulated by virtual setups.	Post-treatment digital models were superimposed with virtual setups using best-fit registration in eModel software (n=23; nonextraction: n=18, extraction: n=5).	- Linear variables (mesiodistal, buccolingual, occlusogingival)<br>- Angular variables (inclination, angulation, rotation)	Mesiodistal and vertical tooth position as well as angular movements (tip and torque) were clinically ideal for nearly all teeth (within 0.5 mm), while buccolingual tooth position was not clinically ideal for seven teeth.	Virtual setups could predict actual treatment outcomes of SureSmile, which could be varied with tooth type and dimension of movement.	B	Moderate
Moreira et al. (2020) [52]	eXceed	To evaluate the accuracy between a CAD-CAM system and actual treatment outcome of non-extraction Class I malocclusion with mild spacing or crowding.	Digital models were exported to eXceed for virtual setups. The virtual setup and actually treated models were measured for the differences with Geomagic Design X (n=24).	Alignments, overjet, marginal ridges, occlusal contact, interproximal contact, occlusal relationship, buccolingual inclination, and rotations	There were statistically significant differences in three out of seven variables. Occlusal contact, interproximal contact, and overjet score of actual outcomes were larger than virtual occlusion although there was no significant difference.	Virtual setup could closely predict the final teeth position with interarch relationships.	B	Moderate
Shantiyai et al. (2020) [46]	uLab	To determine accuracy between virtual setups and actual orthodontic treatment outcomes in Class II division 1 malocclusions with premolar extractions.	Pre-treatment, post-treatment and uLab virtual setups were identified and measured using uLab Systems Software (n=46).	- Intermolar width<br>- Intercanine width<br>- Overjet<br>- Overbite	The measurements of virtual setup were mostly higher than the actual treatment outcome.	Although there were statistically significant discrepancies in overbite and overjet between virtual setups and actual treatments, they were not clinically significant, so this study supported the use of virtual setups in orthodontics.	B	Moderate
Smith et al. (2015) [53]	SureSmile	To investigate the accuracy of the virtual setup and SureSmile treated cases in root	Initial model, virtual setup model, and post-treatment model were measured, and the discrepancies of	- Root inclination<br>- Root angulation	The outcome discrepancies of several teeth were statistically significant, but just a couple of variables	Tipping movements were more predictable with virtual setups, compared to than torque movements in nearly all teeth.	B	Moderate

		inclination and angulation.	variables were compared (n=30).		showed clinical significance.			
de Waard et al. (2022) [47]	OrthoAnalyzer	To examine the accuracy of digital setups in predicting the tooth positions following presurgical orthodontic treatment.	Discrepancies between virtual setups and actual treatment models were examined using linear mixed model analyses (n=26).	- Linear variables (mesiodistal, buccolingual, occlusogingival)<br>- Angular variables (inclination, angulation, rotation)	Clinical differences were found with 44-52% and 74-75% of translations and rotations, respectively.	The accuracy of orthodontic virtual setup in the prediction of pre-surgical orthodontic treatment outcomes was not yet sufficient.	B	Moderate
